# Emergency nursing care of a patient with acute appendicitis complicated by esomeprazole-induced anaphylactic shock: a case report

**DOI:** 10.3389/fsurg.2026.1932010

**Published:** 2026-09-08

**Authors:** Jiangfeng Pu, Li Xin, Yikuan Chen, Xuemei Jiang

**Affiliations:** 1 Gastrointestinal & Anorectal Surgery, The Second Affiliated Hospital of Chongqing Medical University, Chongqing, China; 2 Acute and Critical Care Nursing Innovation and Translation Engineering Research Center of Chongqing Education Commission of China; 3 Chongqing Geriatric Specialized Nursing Clinical Diagnosis and Treatment Center; 4 Department of Vascular Surgery, The Second Affiliated Hospital of Chongqing Medical University, Chongqing, China

**Keywords:** acute appendicitis, anaphylactic shock, emergency care, esomeprazole, target temperature management, veno arterial extracorporeal membrane oxygenation (VA-ECMO)

## Abstract

**Rationale:**

Esomeprazole-induced anaphylactic shock followed by cardiac arrest is an extremely rare but life-threatening adverse event, particularly in patients with acute appendicitis. Clinical nursing experience in managing such critical conditions remains limited, and this case report provides valuable insights into emergency and intensive nursing care.

**Patient concerns:**

A 53-year-old male patient with acute appendicitis developed facial flushing, urticaria, and loss of consciousness within 5 min after starting esomeprazole infusion, rapidly progressing to ventricular fibrillation and cardiac arrest.

**Diagnoses:**

Acute appendicitis complicated by esomeprazole-induced anaphylactic shock and secondary cardiac arrest.

**Interventions:**

Key nursing interventions included rapid identification of allergic manifestations, immediate activation of the rapid response team, cardiopulmonary resuscitation, electrical defibrillation, endotracheal intubation, and timely initiation of Veno arterial extracorporeal membrane oxygenation (VA-ECMO) combined with mechanical ventilation. Nursing care also encompassed development of a safe transport plan under VA-ECMO support, dynamic monitoring of inflammatory markers with anti-infective nursing, precise targeted temperature management using specialized observation charts, close skin monitoring to prevent recurrence of allergic reactions, and multimodal psychological nursing interventions.

**Outcomes:**

After 48 days of intensive treatment and meticulous nursing care, the patient's condition stabilized, and he was successfully discharged with good recovery at one-month follow-up.

**Lessons:**

Rapid recognition of drug allergy, efficient multidisciplinary collaboration, precise VA-ECMO circuit management, systematic temperature control, and comprehensive psychological support are critical to improving survival and neurological outcomes in patients with esomeprazole-induced anaphylactic shock complicated by cardiac arrest.

## 1 Introduction

Acute appendicitis is one of the most common causes of acute abdominal pain in emergency settings. It is characterized by a high incidence and rapid progression, and if not treated promptly, it can easily lead to serious complications such as appendiceal perforation, peritonitis, and even sepsis, posing a significant threat to patient survival (1).

Anaphylactic shock is a severe systemic allergic reaction primarily mediated by immunoglobulin E (Ig E). It can simultaneously affect the skin, respiratory tract, and circulatory system, with rapid disease progression, and may escalate from mild cutaneous manifestations to life-threatening shock within minutes once it occurs (2). Esomeprazole is the levorotatory isomer of omeprazole, consisting of a single S-enantiomer. Owing to its stereoselective metabolism, reduced hepatic first-pass effect, and slower plasma clearance, it exhibits significant pharmacodynamic advantages and has therefore become a commonly used clinical medication (3). Common adverse reactions associated with esomeprazole allergy include diarrhea, dizziness, nausea, vomiting, and abdominal pain (4). However, the incidence of severe adverse events is low, and cardiac arrest caused by allergic reactions is particularly rare. At present, there are relatively few case reports in China on cardiac arrest induced by hypersensitivity to esomeprazole, and clinical nursing experience in managing such cases remains limited. In this case report, we present a patient with acute appendicitis who developed cardiac arrest three minutes after receiving esomeprazole infusion and subsequently required mechanical ventilation and venoarterial extracorporeal membrane oxygenation (VA-ECMO) support. After 48 days of intensive treatment and comprehensive nursing care, the patient achieved recovery of organ function, was able to ambulate, and demonstrated a favorable neurological prognosis. In this case, a favorable patient outcome was achieved through multidisciplinary collaboration and precision nursing interventions. This case provides a valuable reference for nursing care in rare instances of drug-induced anaphylactic shock requiring VA-ECMO support, and the nursing experience is reported as follows.

## 2 Case report

A 53-year-old male patient was admitted to the hospital with a 4-day history of right lower abdominal pain and was diagnosed with acute appendicitis. He had no prior history of drug allergies or cardiac disease. Abdominal computed tomography revealed changes in the appendix and surrounding tissues, consistent with appendicitis. Laboratory examination showed a C-reactive protein level of 15.20 mg/L and a prothrombin activity of 129%.

On the second day of admission, after 3 min of starting the infusion of esomeprazole, the patient developed facial flushing, scattered urticarial wheals on the chest, and pruritus at the affected area, accompanied by symptoms of gastric acid reflux, chest tightness, and dyspnea—indicating involvement of the skin, gastrointestinal, and respiratory systems. Five minutes later, the patient became unresponsive, with trismus, foaming at the mouth, and generalized limb convulsions—suggesting central nervous system involvement due to severe cerebral hypoperfusion. The patient was immediately placed in a supine position without a pillow and administered high-flow oxygen. Esomeprazole-induced drug allergy was suspected. Anti-allergic and anti-shock treatments were administered as prescribed; however, the response was poor. Vital signs revealed a blood pressure of 41/28 mmHg (1 mmHg = 0.133 kPa), heart rate of 46 beats/min, respiratory rate of 14 breaths/min, and oxygen saturation of 88%. Cardiopulmonary resuscitation, electrical defibrillation, and endotracheal intubation were immediately initiated. After approximately 30 min of resuscitation, ventricular fibrillation persisted despite four defibrillation attempts at 200 J. As the patient's vital signs remained unstable, the hospital's VA-ECMO team was urgently consulted to initiate further bedside treatment.

### 2.1 Indications for VA-ECMO initiation

The decision to initiate VA-ECMO was based on the following explicit criteria: (1) refractory ventricular fibrillation despite four defibrillation attempts at 200 J; (2) failure to achieve return of spontaneous circulation (ROSC) after approximately 30 min of continuous cardiopulmonary resuscitation; (3) persistent hemodynamic instability with a nadir blood pressure of 41/28 mmHg despite maximal vasopressor support (norepinephrine infusion); and (4) the patient's age (53 years) and absence of significant comorbidities, suggesting a reasonable likelihood of favorable neurological recovery with mechanical circulatory support. The hospital's ECMO rapid-response team was activated according to institutional protocol for refractory cardiac arrest not responding to standard resuscitation measures.

### 2.2 Cardiovascular evaluation

To exclude alternative cardiac etiologies, the patient underwent bedside transthoracic echocardiography immediately upon ICU admission, which revealed no regional wall motion abnormalities, no pericardial effusion, and preserved left ventricular ejection fraction (approximately 55%–60%). Coronary angiography was not performed during the acute phase due to the patient's hemodynamic instability and ongoing VA-ECMO support, which precluded safe transport to the catheterization laboratory. However, serial electrocardiograms performed throughout the ICU course showed no dynamic ST-segment changes or pathological Q-wave development, and serial cardiac troponin I levels remained within normal limits (<0.04 ng/mL). These findings effectively excluded acute coronary syndrome, Kounis syndrome, and primary myocardial pathology, further supporting anaphylactic shock as the primary etiology with secondary cardiac arrest.

### 2.3 Biomarker assessment

According to EAACI task force recommendations, serum tryptase should be measured 30 min–2 h after symptom onset, with baseline levels obtained at least 24 h after complete symptom resolution. Regrettably, due to the emergent nature of the cardiac arrest and the immediate focus on resuscitation, acute-phase serum tryptase was not obtained during the critical window. A baseline tryptase level was also not measured at 24 h post-recovery, as the patient remained on VA-ECMO support with hemodynamic instability during that period. We acknowledge this as a significant limitation of this case report.

### 2.4 Causality assessment

To objectively evaluate the causal relationship between esomeprazole administration and the anaphylactic reaction, we applied the Naranjo Adverse Drug Reaction Probability Scale (5). The patient scored 7 points (Table 1): previous conclusive reports on this reaction (+1); adverse event appeared after drug administration (+2); adverse event improved after drug discontinuation (+1); no alternative causes identified (+1); reaction confirmed by objective evidence (+1); and no similar reaction to the same drug in previous exposure (+1). A score of 5–8 indicates a probable adverse drug reaction. According to the WHO-UMC causality assessment system, the event had a plausible time relationship to drug intake and could not be explained by the patient's underlying disease or other medications, further supporting a probable causal association.

**Table 1:** Naranjo adverse drug reaction probability scale assessment.

Naranjo Scale Question	Score
1. Previous conclusive reports on this reaction?	+1
2. Did adverse event appear after drug administration?	+2
3. Did adverse reaction improve when drug discontinued?	+1
4. Did adverse reaction reappear on re-administration?	0
5. Alternative causes identified?	+1 (no)
6. Reaction confirmed by objective evidence?	+1
7. Similar reaction to same drug in previous exposure?	+1 (no)
Total	7

**Figure 1:**
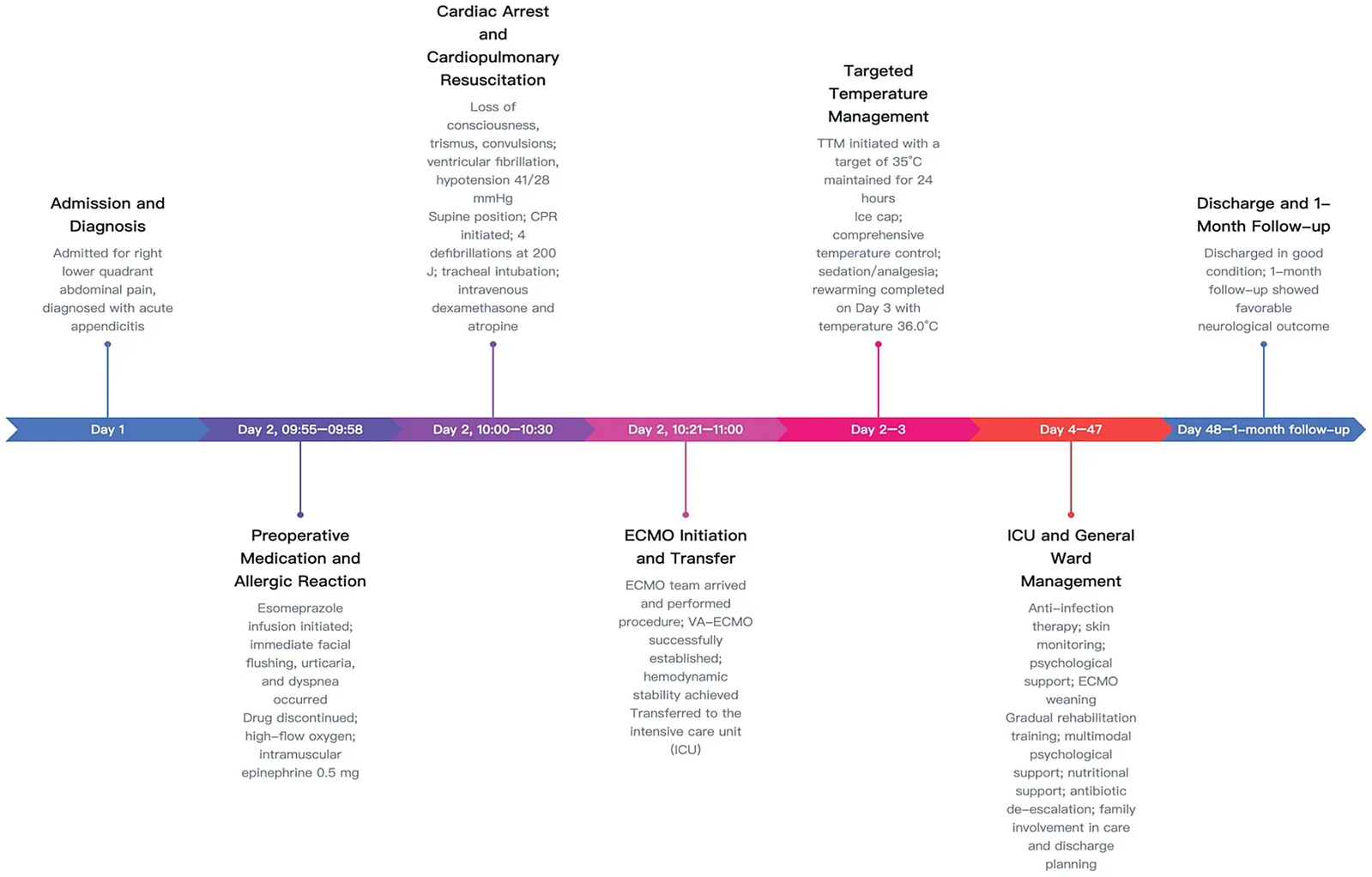
Clinical timeline of the patient's course from admission to one-month follow-up.

Applying the World Allergy Organization (WAO) 2020 diagnostic criteria for anaphylaxis (2), the patient fulfilled Criterion 1: acute onset of illness (within minutes) with involvement of the skin/mucosal tissue (facial flushing and generalized urticaria) and respiratory compromise (dyspnea) and reduced blood pressure or associated symptoms of end-organ dysfunction (hypotension 41/28 mmHg and loss of consciousness). The European Academy of Allergy and Clinical Immunology (EAACI) (6) guidelines similarly emphasize that anaphylaxis is a clinical diagnosis based on recognition of a constellation of presenting features, all of which were present in this case. To provide a clear chronological overview of the patient's clinical course from symptom onset through recovery, a comprehensive timeline of key events was constructed in accordance with the CARE guidelines (7) (Figure 1).

The patient provided written informed consent for the publication of this case report and any accompanying images. This case report was conducted in accordance with the ethical standards of the institutional review board.

## 3 Nursing care

### 3.1 Rapid identification of drug allergy and multidisciplinary rescue with coordinated nursing care

Drug-induced anaphylactic shock has an abrupt onset, with symptoms developing shortly after exposure to the offending allergen in most cases. According to published data (8), approximately 50% of patients develop symptoms within 5 min of allergen exposure, up to 90% within 30 min, and only 10%–20% present with symptoms after more than 30 min. Drug-induced anaphylactic shock has a rapid onset and progression. During drug infusion, if a patient reports any discomfort, nurses should remain highly vigilant, continuously monitor consciousness, heart rate, blood pressure, and oxygen saturation, and closely observe for early signs of hypersensitivity, thereby facilitating early identification and timely intervention.

Allergic reactions induced by esomeprazole may clinically resemble drug stress reactions, arrhythmias, or pseudo allergic responses; however, such reactions typically do not resolve spontaneously, nor can they be alleviated by drug discontinuation or psychological interventions alone. Previous studies have reported that during intravenous esomeprazole infusion, patients may experience stress-related reactions caused by anxiety, pain, or rapid infusion rates, presenting as facial flushing, conjunctival congestion, sweating, chest tightness, blurred vision, numbness of the lips or face, and dyspnea (4). These symptoms usually resolve spontaneously within 3–5 min after drug discontinuation, psychological reassurance, and guided deep breathing.

In the present case, during intravenous infusion of esomeprazole, the patient developed facial flushing, numbness and a tightening sensation of the lips, glabellar area, and bilateral ears, accompanied by nausea with acid reflux, chest tightness, and dyspnea. The patient was immediately placed in the supine position with the head turned to one side, administered high-flow oxygen, and provided with emotional reassurance; the suspected drug was discontinued, the infusion line was replaced, two peripheral intravenous access routes were rapidly established, and vital signs were continuously monitored. As prescribed, intramuscular epinephrine (0.5 mg) was administered immediately. Although intravenous access had been established, the intramuscular route was chosen for initial epinephrine administration in accordance with anaphylaxis treatment guidelines, which recommend it as the first-line therapy due to its superior safety profile, rapid absorption, and ease of administration even in the context of tenuous peripheral circulation. Intravenous epinephrine was reserved for titration if the patient failed to respond to initial intramuscular doses, given the monitored intensive care setting. Subsequently, intravenous dexamethasone (5 mg) and intravenous atropine (0.25 mg) were administered as prescribed; however, symptoms persisted for several minutes without improvement, and the patient developed scattered urticaria with pruritus, fever, profuse sweating, and progressive hypotension (nadir 41/28 mmHg), indicative of shock. Previous studies have reported that anaphylactoid reactions may resemble true allergic reactions clinically but are typically transient and self-limiting (9). In this case, facial flushing occurred within 3 min of infusion and progressed to loss of consciousness within 5 min; based on the clinical course and manifestations, stress reactions and anaphylactoid reactions were excluded, leading to a rapid diagnosis of esomeprazole-induced anaphylactic shock.

Based on the drug allergy early warning score, the nurse immediately activated the hospital Rapid Response Team (RRT). The RRT is designed to rapidly identify clinical deterioration in general ward patients and to implement timely early interventions. The effectiveness of the RRT depends on healthcare staff's ability to recognize early warning signs, prompt team activation, and rapid implementation of interventions, which collectively contribute to reducing the incidence of cardiac arrest and in-hospital mortality.

Based on the medication history, intravenously administered esomeprazole, a proton pump inhibitor, was identified as the most likely allergen. According to medical orders, sodium dexamethasone phosphate, epinephrine, calcium gluconate, and norepinephrine were immediately administered for anti-allergic and anti-shock treatment, with close continuous monitoring. As ventricular fibrillation and hypotension could not be corrected, a multidisciplinary rescue was initiated involving the intensive care unit, respiratory medicine, vascular surgery, and ultrasonography teams, who jointly evaluated the patient and decided to proceed with invasive mechanical ventilation combined with extracorporeal membrane oxygenation (ECMO) support. The hospital's mechanical ventilation and ECMO rapid response teams were immediately summoned to the bedside. At 10:00, the responsible nurse initiated the process via the hospital's ECMO rapid-response intelligent collaboration system, which monitors and records six key time points-initiation of preparation, completion of preparation, departure, arrival, cannulation, and initiation of ECMO-providing objective data to shorten ECMO setup time and improve rescue efficiency. The ECMO team completed equipment preparation at 10:05 and arrived at the ward at 10:07. Subsequently, one physician communicated with the patient's family and obtained informed consent, while another simultaneously performed skin preparation and disinfection. Nurses were responsible for checking the operating environment, arranging equipment priming, connecting pipelines and gas sources, assisting with cannulation, and orderly passing instruments. The procedure officially commenced at 10:21, and venoarterial ECMO (VA-ECMO) was successfully established at 10:41, resulting in hemodynamic stabilization. This system effectively shortened the time required for ECMO initiation and significantly improved resuscitation efficiency.

### 3.2 A comprehensive transport nursing plan was developed to ensure safe transfer under extracorporeal membrane oxygenation (ECMO) combined with mechanical ventilation support

In this case, the patient was in the post–cardiac arrest syndrome phase, with significant hemodynamic instability and multiple indwelling therapeutic catheters. Vibration and movement during transport could exacerbate circulatory instability and increase the risk of unplanned device dislodgement, posing a major challenge to the nursing team.

Therefore, a detailed transport plan was developed in advance, with thorough discussion and preparation for potential emergencies that might occur during transfer. Prior to transfer, the optimal route was comprehensively evaluated, and advance communication with the ICU was conducted to confirm the availability and proper functioning of the bed unit, ventilator, and ECMO workstation, including power supply and gas interfaces, device battery levels and operational status, as well as the completeness of emergency medications and equipment. Coordination was also carried out in advance with relevant departments and elevator dispatch personnel to ensure seamless transfer. In addition, communication with the patient's family was completed, and the responsibilities and observation priorities of each transport team member were clearly defined. During transport, roles were clearly assigned: one nurse was responsible for monitoring the ECMO system and cannulas; one nurse managed the ventilator and airway; one nurse monitored the patient monitor, infusion pumps, and central venous lines while ensuring secure fixation; and one nurse supervised smooth and steady movement of the transport bed. Meanwhile, one physician continuously assessed the patient's clinical condition, and another physician was responsible for overall coordination and guidance. Upon arrival at the ICU, the patient was properly positioned, vital signs and hemodynamic status were reassessed, and all devices were confirmed to be functioning normally with securely fixed and intact lines. Ultimately, no significant hemodynamic fluctuations or unplanned device dislodgements occurred during transport from the ward to the ICU.

### 3.3 Dynamic monitoring of inflammatory markers and implementation of targeted anti-infective nursing care, with emphasis on ECMO cannula site management

Continuous monitoring showed a white blood cell count of 14.98 × 10⁹/L, a neutrophil percentage of 90.4%, and a lactate level of 5.7 mmol/L. While infection may have contributed, the elevated lactate was more likely attributable to severe anaphylactic shock and subsequent circulatory collapse. Antibiotic therapy (initially piperacillin-tazobactam and vancomycin, later de-escalated based on culture results) was administered as ordered. Crucially, given the patient's immunocompromised state post-arrest and the presence of invasive devices including ECMO cannulas, nursing care focused on preventing healthcare-associated infections, particularly cannula-related bloodstream infections. Specific nursing protocols for the VA-ECMO cannula sites included:

Site inspection and dressing changes: The arterial (femoral) and venous (femoral) cannulation sites were inspected every 2 h for signs of bleeding, hematoma, infection (erythema, warmth, purulence), or limb ischemia (color, pulse, temperature, and capillary refill time of the ipsilateral leg). Sterile, transparent, semipermeable dressings were used, and dressing changes were performed every 48 h or immediately if soiled or loose, using a strict aseptic technique.
Securement and dislodgement prevention: The cannulas were secured using a standardized suture bridge and transparent film dressing. The tubing was carefully anchored to prevent traction or accidental dislodgement during patient positioning or transport. The position of the cannulas was documented with photographs at the time of placement and reassessed each shift.
Circuit monitoring for infection clues: Signs of systemic inflammation were correlated with circuit parameters. Any persistent fever, rigors, or leukocytosis prompted blood cultures drawn from both peripheral sites and the circuit, and the cannula sites were emergently re-evaluated.

In addition, protective isolation was implemented in a single room with restricted access, dedicated nursing staff, and daily environmental disinfection. Through these interventions, no cannula-related infections or other nosocomial infections occurred throughout the 48-day ICU course.

### 3.4 Precise targeted temperature management was carried out, and nursing observation charts were used to prevent complications

Targeted temperature management (TTM) is a clinical intervention that employs physical and pharmacological methods to rapidly reduce core body temperature to a predetermined target, maintain it for a specified duration, and subsequently allow a controlled, gradual rewarming to the normal range while preventing temperature fluctuations (10). This intervention reduces cerebral metabolic demand, alleviates cerebral edema, and decreases ischemia-hypoxia-induced neuronal apoptosis and necrosis, thereby improving survival rates and neurological outcomes in patients after cardiac arrest.

Given that effective resuscitation may lead to reperfusion-induced hyperthermia and exacerbation of cerebral edema, mild hypothermia therapy was initiated as early as possible after cardiac arrest. An ice cap was applied to the patient's head within 3 min of cardiac arrest onset. After VA-ECMO establishment, the integrated temperature control system was used to stabilize the patient's core temperature at 35 °C within 1 h. Continuous infusion of midazolam (1 mg/h) and remifentanil (0.1 mg/h) was administered for sedation and analgesia. Nursing care included hourly documentation of vital signs and clinical parameters using a standardized observation chart, including temperature, heart rate, rhythm, blood pressure, urine output, skin color, and pupillary light reflex. Routine interventions, such as repositioning, chest percussion, and airway humidification, were performed to prevent pulmonary infection. Coagulation function was closely monitored via APTT and platelet counts, and serum potassium and sodium levels were tracked to prevent electrolyte disturbances.

In addition, because the patient was supported by VA-ECMO, bedside nurses performed specialized ECMO circuit monitoring and management as follows:

Hemodynamic and flow parameters: Hourly documentation of VA-ECMO blood flow rate (maintained at 3.5–4.0 L/min), revolutions per minute (RPM, typically 3,000–3,500), sweep gas flow rate (2–4 L/min, adjusted to maintain PaCO₂ 35–45 mmHg), and membrane oxygenator gradient pressure (normally < 20 mmHg; an increasing trend suggests oxygenator thrombosis). These parameters were trended over time to detect early signs of circuit or patient instability.
Identification of circuit “chatter” and preload deficiency: The ECMO circuit was continuously inspected for chatter (a rhythmic oscillation of the venous line and tubing), which indicates inadequate preload. In the setting of post-anaphylactic distributive shock, intravascular volume depletion is common and can precipitate flow instability. When chatter was observed, the nurse immediately notified the attending physician and assisted in volume loading or temporary pump speed adjustment to restore stable flow.
Surveillance for circuit complications: Every hour, the entire ECMO circuit (cannulas, tubing, oxygenator, and connector sites) was visually inspected for visible clots, air bubbles, or signs of oxygenator failure (e.g., worsening gas exchange despite unchanged sweep gas). Signs of hemolysis—such as dark or pink-tinged plasma in the circuit, rising plasma free hemoglobin levels, or sudden hemoglobinuria—were also monitored. Any abnormality was documented and communicated without delay.
Structured communication protocol: A standardized SBAR (Situation, Background, Assessment, Recommendation) communication tool was used whenever a nurse identified an ECMO-related issue (e.g., chatter, clot, bleeding at cannulation site, or unexpected changes in flow or pressure). The bedside nurse reported directly to the intensivist or the perfusionist, and a daily multidisciplinary ECMO round was conducted, during which the nurse presented the previous 24 h of circuit parameters, cannula site status, and any alarm events. A multidisciplinary expert communication group was also established, and monitoring data were uploaded hourly to facilitate real-time adjustments to the treatment plan.

After 24 h of targeted temperature management, the patient's core temperature was gradually restored to 36.0 °C at a rate of 0.1 °C per hour.

### 3.5 Close dynamic monitoring of the skin was conducted to prevent recurrence of allergic reactions

Cutaneous manifestations are common early clinical signs of drug allergy. In this case, esomeprazole had already triggered severe anaphylactic shock, and during ICU treatment the patient's immune system remained in a highly sensitized state, posing a substantial risk of re-exposure to allergens or cross-reactive reactions. Therefore, the skin was regarded as a critical monitoring window, and proactive, close dynamic observation was considered essential.

The specific nursing plan included establishing a systematic skin surveillance protocol. In addition to routine vital sign monitoring, a “Drug Allergy Skin Monitoring Record” was implemented, with hourly assessments documenting and evaluating facial, neck, trunk, and limb areas for erythema, urticaria, and angioedema. Clear observation priorities and documentation standards were defined, focusing on the presence of new-onset or progressive erythema, urticaria, angioedema (particularly involving the eyelids and lips), pruritus, diaphoresis, or pallor, with detailed descriptions of lesion morphology, distribution, color, and progression.

An immediate response protocol was also established, whereby any suspicious skin change was regarded as a potential allergic warning signal, prompting immediate review of all intravenous and enteral medications, blood products, and contact materials; suspension of non-essential drugs; prompt notification of physicians; and preparation for initiation of anti-allergic treatment (e.g., epinephrine and glucocorticoids). This systematic skin monitoring and early-warning framework aimed to facilitate early identification and timely management of allergic reactions, thereby preventing recurrence of life-threatening hypersensitivity events.

### 3.6 Multimodal psychological nursing interventions were provided

The patient successfully recovered from cardiac arrest but remained at high risk of sudden death. During the period of regained consciousness, he exhibited intense fear as well as a strong survival instinct. The healthcare team implemented structured psychological interventions, which included clearly explaining the treatment plan and prognosis to alleviate fear; avoiding bedside handovers to reduce psychological burden; providing counseling to family members by explaining the resuscitation process and expected outcomes, answering questions, and guiding them to offer emotional support to the patient via video calls, thereby preventing the transmission of anxiety from family to patient.

Simultaneously, professional, and gentle nursing care, combined with comprehensive rehabilitation and nutritional guidance, was provided to gradually build patient trust, enabling him to actively cooperate with treatment and recovery in a positive and stable psychological state.

## 4 Discussion

### 4.1 Differential diagnosis

Several alternative etiologies of sudden cardiovascular collapse in this patient warrant consideration. Kounis syndrome (11), defined as acute coronary syndrome triggered by an allergic or anaphylactic reaction, should be considered in any patient presenting with acute coronary events following allergen exposure. In this case, although the patient presented with hypotension and cardiac arrest, bedside transthoracic echocardiography performed upon ICU admission revealed no regional wall motion abnormalities, no pericardial effusion, and preserved left ventricular ejection fraction (approximately 55%–60%). Serial electrocardiograms performed throughout the ICU course showed no dynamic ST-segment changes or pathological Q-wave development, and serial cardiac troponin I levels remained within normal limits (<0.04 ng/mL). Coronary angiography was not performed during the acute phase due to the patient's hemodynamic instability and ongoing VA-ECMO support, which precluded safe transport to the catheterization laboratory; however, the combination of normal echocardiographic findings, lack of dynamic ECG changes, and normal serial troponin levels effectively excluded acute coronary syndrome, Kounis syndrome, and primary myocardial pathology. The absence of echocardiographic wall motion abnormalities, normal serial troponin levels, and lack of dynamic ECG changes further support the exclusion of Kounis syndrome, which requires evidence of acute myocardial ischemia (ECG changes, elevated cardiac biomarkers, or wall motion abnormalities) in the setting of an allergic reaction (11). Acute coronary syndrome (ACS) due to atherosclerotic plaque rupture or coronary vasospasm was also considered; however, the patient had no prior history of cardiac disease, no typical ischemic chest pain (the patient was unconscious shortly after symptom onset), and the above cardiac evaluation findings did not support acute myocardial ischemia. Primary malignant arrhythmias such as Brugada syndrome or long QT syndrome were also considered; however, the patient had no family history of sudden cardiac death, no prior syncopal episodes, and a normal baseline ECG prior to admission. The temporal relationship between esomeprazole infusion and symptom onset, combined with the classic cutaneous manifestations of anaphylaxis (flushing and urticaria) and the exclusion of alternative cardiac etiologies through comprehensive cardiovascular evaluation, strongly supports anaphylactic shock as the primary etiology, with ventricular fibrillation as a secondary consequence of severe hypotension and myocardial hypoperfusion rather than a primary arrhythmic event.

### 4.2 Anaphylactic shock vs. septic shock

The presence of fever in this case warrants careful consideration of septic shock as an alternative diagnosis. Several factors argue against septic shock as the primary etiology. First, the onset of symptoms occurred within 3 min of esomeprazole infusion, which is too rapid for a bacterial infection to manifest. Second, the classic cutaneous manifestations of anaphylaxis (flushing and urticaria) preceded the hemodynamic collapse, which is not a feature of septic shock. Third, the patient's C-reactive protein level on admission was only 15.20 mg/L, which is not indicative of severe sepsis. Fourth, blood cultures obtained during the acute event and throughout the ICU stay remained negative. The fever observed during the ICU course (maximum 38.5 °C) was more likely attributable to post-cardiac arrest syndrome—a systemic inflammatory response following ischemia-reperfusion injury—rather than infection. This is supported by the fact that the fever resolved with targeted temperature management and did not recur after rewarming, without specific anti-infective therapy targeting the fever itself. While the patient did receive empirical antibiotics (piperacillin-tazobactam and vancomycin) given the immunocompromised state post-arrest and the presence of invasive devices, these were administered as prophylactic measures rather than treatment for confirmed sepsis and were subsequently de-escalated based on negative culture results.

### 4.3 Evaluation for clonal mast cell disorders

The patient had no prior history of drug allergies, which raises the question of whether an underlying clonal mast cell disorder (MCD) may have predisposed him to such a severe reaction. In patients with idiopathic or severe anaphylaxis, evaluation for a clonal MCD is recommended, including measurement of baseline serum tryptase, testing for the KIT p.D816 V mutation in peripheral blood using high-sensitivity assays, and calculation of a mast cell clonality prediction score (12). A bone marrow biopsy should be performed in patients with elevated baseline tryptase or a high pretest probability of clonal MCD. In this case, although the severity of the reaction and the absence of prior allergies could theoretically suggest an underlying mast cell disorder, the patient had no cutaneous stigmata of mastocytosis (e.g., urticaria pigmentosa), no history of recurrent anaphylaxis, and no gastrointestinal or constitutional symptoms suggestive of systemic mastocytosis. Baseline tryptase was not measured (as noted above), and the patient declined further allergological workup after discharge. Nevertheless, we have recommended that the patient undergo evaluation for clonal MCD, including serum tryptase and KIT D816 V testing, during follow-up.

### 4.4 Allergological follow-up and cross-reactivity considerations

Following hospital discharge, the patient was referred to the Department of Allergy and Clinical Immunology for comprehensive allergological evaluation. Skin testing (skin prick test and intradermal test) with esomeprazole and other proton pump inhibitors (PPIs) was recommended to confirm the causative agent. The basophil activation test (BAT) (13), which has been shown to be a reliable option in the diagnosis of immediate hypersensitivity to PPIs, was also considered. However, the patient declined further diagnostic testing due to anxiety regarding potential re-exposure.

The basophil activation test (BAT) is a flow cytometry-based *in vitro* assay that assesses basophil activation (via surface markers such as CD63) upon allergen stimulation. In the diagnosis of immediate hypersensitivity to PPIs, BAT offers several advantages: it is an *in vitro* test with no risk of inducing systemic reactions in sensitized patients, making it particularly valuable when skin testing is negative or contraindicated, and when drug provocation testing is considered too risky. Previous studies have demonstrated that BAT has a sensitivity of approximately 74% for diagnosing omeprazole hypersensitivity, with 100% specificity and positive predictive value (14). Importantly, when combined with skin testing, BAT can increase the overall diagnostic sensitivity to approximately 86%, and it can be positive in up to 57% of patients with negative skin tests. However, BAT also has limitations: it requires fresh whole blood and must be processed within 24 h of collection, it is technically demanding and requires specialized flow cytometry equipment and expertise, and its sensitivity may decrease over time after the index reaction. Nevertheless, the EAACI recognizes BAT as a reliable option in the diagnosis of immediate hypersensitivity to PPIs. In the present case, BAT was recommended but declined by the patient due to anxiety regarding potential re-exposure—a limitation acknowledged in our study.

Regarding cross-reactivity among PPIs, previous studies have demonstrated that allergic reactions to one PPI do not necessarily indicate cross-reactivity to others; however, given the severity of this patient's reaction, we strongly advised against the use of any PPI in the future and recommended alternative acid-suppressive therapy (e.g., H₂-receptor antagonists) if clinically indicated. The patient was provided with a drug allergy alert card and instructed to inform all healthcare providers of this severe adverse reaction.

### 4.5 Comparison with previously reported cases

PPI-induced anaphylaxis is rare, and cases involving cardiac arrest or requiring mechanical circulatory support are exceedingly uncommon. Kepil Özdemir et al. reported a retrospective series of 42 patients with PPI hypersensitivity, among whom the most common clinical manifestations were urticaria and angioedema; none of the patients in their cohort developed cardiac arrest or required ECMO support (9). Zheng et al. analyzed 418 adverse drug reaction reports related to esomeprazole in China and found that severe anaphylactic reactions accounted for a very small proportion, with no cases of cardiac arrest documented in their series (4). To our knowledge, only isolated case reports have described esomeprazole-induced anaphylactic shock, and cases requiring VA-ECMO support for PPI-induced anaphylaxis are exceptionally rare in the literature. This case therefore provides a unique contribution to the limited body of evidence on the nursing management of severe PPI-induced anaphylaxis complicated by cardiac arrest and VA-ECMO support.

To better contextualize the rarity of the present case, we have summarized previously reported cases of PPI-induced anaphylaxis, particularly those involving cardiac arrest or mechanical circulatory support (15) (Table 2).

**Table 2:** Summary of reported cases of PPI-induced anaphylaxis with cardiac arrest or mechanical circulatory support.

Study/Year	PPI Agent	Patient Age/Sex	Clinical Presentation	Cardiac Arrest	Mechanical Support	Outcome
Kepil Özdemir et al., 2016 (9)	Various PPIs	Mixed (*n* = 42)	Urticaria, angioedema	None	None	Recovered
Zheng et al., 2018 (4)	Esomeprazole	Mixed (*n* = 418 ADR reports)	Various adverse reactions	None reported	None	Variable
Li et al., 2020 (3)	Esomeprazole	Not specified	Anaphylactic shock	Not specified	Not specified	Recovered
Yuan et al., 2023 (15)	Various PPIs	Mixed (*n* = 84)	Anaphylactic shock	few such cases	Not specified	Variable
Present case, 2026	Esomeprazole	53-year-old male	Anaphylactic shock, VF, cardiac arrest	**Yes**	**VA-ECMO**	**CPC 1, mRS 1**

Bold values indicate the distinctive features of the present case compared with previously reported cases. The present case is the only one among the summarized reports that involved both cardiac arrest and VA-ECMO support. VF, ventricular fibrillation; CPC, Cerebral Performance Category; mRS, modified Rankin Scale; ADR, adverse drug reaction.

### 4.6 Neurological outcome assessment

The patient's neurological recovery was objectively evaluated using the Cerebral Performance Category (CPC) scale (16). At the time of hospital discharge, the patient achieved a CPC score of 1 (good cerebral performance: conscious, alert, able to work, and lead a normal life, with only minor neurological or psychological deficits). At the one-month follow-up, the CPC score remained 1, indicating favorable neurological recovery. The modified Rankin Scale (mRS) score was 1 at discharge (no significant disability despite some symptoms; able to carry out all usual duties and activities), confirming a good functional outcome.

## 5 Limitations

This case report has several limitations. First, as a single-case study, the findings and nursing experience may not be generalizable to other patients with esomeprazole-induced anaphylactic shock or similar critical conditions, given the rarity of this adverse reaction and the unique clinical context.

Second, the patient had no prior history of drug allergies, which limits the ability to predict such severe reactions in advance, and the absence of confirmatory allergy testing (e.g., skin testing or drug provocation testing) to definitively establish esomeprazole as the causative agent is a limitation, although the temporal relationship and clinical course strongly supported the diagnosis. Confirmatory allergological testing—including skin testing and the basophil activation test (BAT), which has been validated for diagnosing immediate hypersensitivity to PPIs—was recommended but declined by the patient due to anxiety regarding potential re-exposure. This limits the definitive identification of esomeprazole as the causative agent and precludes assessment of cross-reactivity with other PPIs.

Third, the intensive nursing interventions, including VA-ECMO management and targeted temperature management, were performed in a highly specialized tertiary hospital with a well-established rapid response system, which may restrict the applicability of these protocols to resource-limited settings.

Fourth, serum tryptase—both acute-phase and baseline—was not measured. The EAACI guidelines recommend obtaining serum tryptase 30 min–2 h after symptom onset and baseline levels at least 24 h after symptom resolution to support the diagnosis of anaphylaxis. However, the immediate life-threatening nature of the cardiac arrest and the subsequent VA-ECMO deployment precluded timely sample collection. This limits the biomarker confirmation of anaphylaxis.

Fifth, while multimodal psychological nursing was provided, standardized psychological assessment scales were not utilized to quantify the patient's emotional status before and after intervention.

Sixth, the long-term outcomes, including the patient's complete neurological recovery, quality of life, and potential for post-discharge allergic recurrence, were only followed up for one month; extended follow-up is needed to fully assess the durability of recovery and late complications.

Future multicenter studies with larger sample sizes and prospective designs are warranted to validate and refine the nursing strategies described in this report.

## 6 Conclusion

This case report describes the emergency and intensive nursing care of a patient with esomeprazole-induced anaphylactic shock complicated by cardiac arrest, who was successfully managed with VA-ECMO support and comprehensive intensive care. Based on our observational experience in this single case, several nursing practices appeared to contribute to the favorable outcome: rapid recognition of allergic manifestations and timely activation of the rapid response team; meticulous VA-ECMO circuit management with structured interdisciplinary communication; systematic skin monitoring to detect early signs of recurrent allergic reactions; and multimodal psychological support. However, given the inherent limitations of a single case report, these observations should be interpreted as hypothesis-generating insights rather than definitive evidence of efficacy. The nursing strategies described herein may serve as a reference for clinical practice in similar rare scenarios, but their generalizability requires validation through future multicenter studies with larger sample sizes and prospective designs. Rapid recognition of anaphylaxis, efficient multidisciplinary collaboration, and precise critical care nursing remain essential components in improving survival and neurological outcomes in patients with this rare but life-threatening condition.

During the initial resuscitation phase, nurses must possess rapid and accurate emergency judgment skills as well as efficient team collaboration. In the subsequent treatment phase, refined and systematic nursing management is required, significantly increasing the overall complexity of care. Rapid recognition of the patient's condition by nursing staff and timely intervention are critical for improving the success rate of resuscitation. The ECMO team promptly performed bedside cannulation, providing essential support for maintaining vital signs and reversing critical status. During ICU transfer, the development and execution of a comprehensive transport plan ensured patient safety. In subsequent care, meticulous nursing management of the VA-ECMO circuit, including hemodynamic monitoring, infection prevention at cannula sites, and structured interdisciplinary communication, was critical for maintaining circulatory support and avoiding complications. Individualized monitoring using a drug-allergy skin monitoring record effectively prevented recurrence of allergic reactions. The entire treatment process focused not only on life preservation but also on promoting recovery of organ and neurological function, laying an important foundation for the patient's eventual rehabilitation and return to society.

## Data Availability

The raw data supporting the conclusions of this article will be made available by the authors, without undue reservation.

## References

[1] Diaz JJ Ceresoli M Herron T Coccolini F. Current management of acute appendicitis in adults: what you need to know. J Trauma Acute Care Surg. (2025) 98(2):181–9. 10.1097/TA.0000000000004471 39504344

[2] Cardona V Ansotegui IJ Ebisawa M El-Gamal Y Fernandez Rivas M Fineman S. World allergy organization anaphylaxis guidance 2020. World Allergy Organ J. (2020) 13(10):100472. 10.1016/j.waojou.2020.100472 33204386 PMC7607509

[3] Cardona V Ansotegui IJ Ebisawa M El-Gamal Y Fernandez Rivas M Fineman S. One case of anaphylactic shock caused by intravenous infusion of esomeprazole. J Clin Ration Drug Use. (2020) 1:39–41. 10.1016/j.waojou.2020.100472

[4] Zheng FF Chen HD Wang ML. Analysis of 418 case of adverse drug reaction induced by esomeprazole. Chin Hosp Pharm J. (2018) 9:975–8. 10.13286/j.cnki.chinhosppharmacyj.2018.09.17

[5] Naranjo CA Busto U Sellers EM Sandor P Ruiz I Roberts EA. A method for estimating the probability of adverse drug reactions. Clin Pharmacol Ther. (1981) 30(2):239–45. 10.1038/clpt.1981.154 7249508

[6] Muraro A Worm M Alviani C Cardona V DunnGalvin A Garvey LH. EAACI Guidelines: anaphylaxis (2021 update). Allergy. (2022) 77(2):357–77. 10.1111/all.15032 34343358

[7] Gagnier JJ Kienle G Altman DG Moher D Sox H Riley D. The CARE guidelines: consensus-based clinical case reporting guideline development. Glob Adv Health Med. (2013) 2(5):38–43. 10.7453/gahmj.2013.008 24416692 PMC3833570

[8] Zhang WW. Emergency Internal Medicine (4th Edition). Beijing: People's Medical Publishing House (2017).

[9] Kepil Özdemir S Öner Erkekol F Ünal D Büyüköztürk S Gelincik A Dursun AB. Management of hypersensitivity reactions to proton pump inhibitors: a retrospective experience. Int Arch Allergy Appl Immunol. (2016) 171(1):54–60. 10.1159/000450952 27838693

[10] Hypothermia after Cardiac Arrest Study Group. Mild therapeutic hypothermia to improve the neurologic outcome after cardiac arrest. N Engl J Med. (2002) 346(8):549–56. 10.1056/NEJMoa012689 11856793

[11] Kounis NG Koniari I Soufras GD Patsouras N Hahalis G. Kounis syndrome: a review article on epidemiology, diagnostic findings, management and complications of allergic acute coronary syndrome: mastocytosis and post-mortem diagnosis. Int J Cardiol. (2017) 242:38. 10.1016/j.ijcard.2017.02.144 28619339

[12] Navarro-Navarro P González-Tablas M Pérez-Pons A Sánchez-Muñoz L Henriques A Álvarez-Twose I. Improved diagnostic screening and classification of clonal mast cell diseases by ultrasensitive KIT p.D816 V detection. Blood. (2025) 146(22):2696–709. 10.1182/blood.2025029507 40811860 PMC12883851

[13] Laguna JJ Bogas G Salas M Mayorga C Dionicio J Gonzalez-Mendiola R. The basophil activation test can be of value for diagnosing immediate allergic reactions to omeprazole. J Allergy Clin Immunol Pract. (2018) 6(5):1628–1636.e2. 10.1016/j.jaip.2017.12.001 29339127

[14] Fernandez-Santamaria R Salas M Ariza A Jiménez MA González-Mendiola MR Sanchez ML. Basophil activation test positivity decreases with time in immediate allergic reactions to proton pump inhibitors. Allergy. (2025) 80(4):1111–4. 10.1111/all.16406 39601607 PMC11969308

[15] Yuan L Cui JG Su R. Literature analysis of 84 cases of anaphylactic shock caused by proton pump inhibitors. Drugs and Clinic. (2023) 38(6):1494–502. 10.7501/j.issn.1674-5515.2023.06.035

[16] Rankin J. Cerebral vascular accidents in patients over the age of 60: iI. Prognosis. Scott Med J. (1957) 2(5):200–15. 10.1177/003693305700200504 13432835

